# An EAPC white paper on multi-disciplinary education for spiritual care in palliative care

**DOI:** 10.1186/s12904-019-0508-4

**Published:** 2020-01-15

**Authors:** Megan Best, Carlo Leget, Andrew Goodhead, Piret Paal

**Affiliations:** 1 Senior Lecturer, Institute for Ethics and Society, University of Notre Dame, Fremantle, Australia; 2 Post-doctoral research fellow, PoCoG and Sydney Health Ethics, University of Sydney, PO Box, 944, Broadway NSW 2007, Sydney, Australia; 3 Professor in Care Ethics at the University of Humanistic Studies, Kromme Nieuwegracht 29, Utrecht, 3512 HD The Netherlands; 4 Spiritual Care Lead, St Christopher’s Hospice, 51/59 Lawrie Park Road, London, Sydenham SE26 6DZ UK; 5 Researcher at the Palliative Care Research Hub, Institute of Nursing Science and Practice, Paracelsus Medical Private University, Strubergasse 21, 5020 Salzburg, Austria

**Keywords:** Spirituality, Spiritual care, Education, Palliative care, Health care professionals, Existential needs, Religious needs, Spiritual needs, Curriculum, Holistic caregiving, Spiritual assessment

## Abstract

**Background:**

The EAPC White Paper addresses the issue of spiritual care education for all palliative care professionals. It is to guide health care professionals involved in teaching or training of palliative care and spiritual care; stakeholders, leaders and decision makers responsible for training and education; as well as national and local curricula development groups.

**Methods:**

Early in 2018, preliminary draft paper was written by members of the European Association for Palliative Care (EAPC) spiritual care reference group inviting comment on the four core elements of spiritual care education as outlined by Gamondi et al. (2013) in their paper on palliative care core competencies. The preliminary draft paper was circulated to experts from the EAPC spiritual care reference group for feedback. At the second stage feedback was incorporated into a second draft paper and experts and representatives of national palliative care organizations were invited to provide feedback and suggest revisions. The final version incorporated the subsequent criticism and as a result, the Gamondi framework was explored and critically revised leading to updated suggestions for spiritual care education in palliative care.

**Results:**

The EAPC white paper points out the importance of spiritual care as an integral part of palliative care and suggests incorporating it accordingly into educational activities and training models in palliative care. The revised spiritual care education competencies for all palliative care providers are accompanied by the best practice models and research evidence, at the same time being sensitive towards different development stages of the palliative care services across the European region.

**Conclusions:**

Better education can help the healthcare practitioner to avoid being distracted by their own fears, prejudices, and restraints and attend to the patient and his/her family. This EAPC white paper encourages and facilitates high quality, multi-disciplinary, academically and financially accessible spiritual care education to all palliative care staff.

## Background

Palliative care services and interest in palliative care provision are growing across Europe [1], and while not all countries have yet developed full palliative care services with multi-disciplinary teams [2], this paper summarises a shared vision of multi-disciplinary spiritual care (SC) provision in palliative care, for which all services should aim. The content can be incorporated into existing palliative care curricula or used alone to supplement training for palliative care workers who would like further opportunities to improve their competencies in addressing and incorporating spirituality and SC into their everyday practice. Details may need to be adapted according to the setting, and care by referral may be required while palliative care services grow in SC competency. Grief and Bereavement programmes may have spiritual content.

The United Nations (UN) and World Health Organisation (WHO) state that providing access to “palliative care is an ethical responsibility of health care systems, and that it is the ethical duty of health care professionals to alleviate pain and suffering, whether physical, psychosocial or spiritual, irrespective of whether the disease or condition can be cured, and that end-of-life care for individuals is among the critical components of palliative care” [3]. The WHO defines palliative care as a process involving ‘early identification and impeccable assessment and treatment of pain and other problems, physical, psychosocial and spiritual’ [4]. The WHO and UNICEF’s *Astana 2018 Declaration* states that “palliative care must be accessible to all”, and in order to build sustainable primary healthcare, all member states have committed to “prioritize disease prevention and health promotion and will aim to meet all people’s health needs across the life course through comprehensive preventive, promotive, curative, rehabilitative services and palliative care” [5]. Thus, providing palliative care is increasingly recognized as a universal responsibility [6].

As seen in the definitions of palliative care above, the spiritual domain is, and has always been, integral to palliative care. This was established by the founder of modern palliative care, Dame Cicely Saunders, when she identified the multi-dimensional spiritual suffering at the end of life which came to be known as ‘Total Pain’ [7, 8]. As such, spirituality has always been addressed to some extent within palliative care [7, 9]. More recently, there have been international efforts to improve spiritual care not only in palliative care, but in healthcare generally [10, 11]. However, healthcare professionals still report difficulty in grasping what is meant by spirituality and spiritual care and hence often fail to meet the spiritual needs of patients [12–14]. This is a matter of concern as, according to the WHO, the spiritual dimension is an integral meaning-giving aspect of human existence and spiritual needs are commonly experienced by patients with sudden ill-health or loss, chronic conditions, and life-limiting conditions [15–24].

Building on work emerging from the USA [10, 11], the EAPC Spiritual Care reference group, a former taskforce,  was formed in 2010 to improve SC provision in Europe, taking into account the diversity across the continent [12]. This EAPC White Paper has been written to provide guidance on SC education for all health care workers who provide palliative care, regardless of discipline and care context. The target groups for this paper include: [1] Health care workers involved in teaching or training palliative care and SC [2]; Stakeholders, leaders and decision makers responsible for training and education provided to all healthcare workers involved in palliative care; and [3] National and local curricula development groups. SC involves recognising the importance of the spiritual dimension for patients and caregivers struggling with health- or death-related crises, chronic conditions or life-limiting illness and involves needs assessment and provision of support. Provision of SC in palliative care is important not only for patients and families/caregivers, but also for healthcare workers [25]. Engagement with spirituality enriches the lives of all involved; those who give and receive care, including children [26]. The level of unmet need in SC across Europe is not clear, and variable levels of provision are reported [15, 27, 28].

Spirituality is a universal dimension of human beings, and therefore all patients will benefit from appropriate SC. For many people, who engage in SC provision, it is done intuitively by understanding and connecting to patients and caregivers as a human act, and something that might be difficult to capture in words. In order to operationalise SC it is helpful for all palliative care providers to be familiar with the EAPC working definition of spiritual care:

‘Spirituality is the dynamic dimension of human life that relates to the way persons (individual and community) experience, express and/or seek meaning, purpose and transcendence, and the way they connect to the moment, to self, to others, to nature, to the significant and/or the sacred.’

Spirituality is multidimensional, consisting of 1. Existential challenges (e.g. questions concerning identity, meaning, suffering and death, guilt and shame, reconciliation and forgiveness, freedom and responsibility, hope and despair, love and joy). 2. Value based considerations and attitudes (what is most important for each person, such as relations to oneself, family, friends, work, things, nature, art and culture, ethics and morals, and life itself). 3. Religious considerations and foundations (faith, beliefs and practices, the relationship with God or the ultimate). However, it is critical to comprehend that in care situations it is the patient who tells us the form their own spirituality takes. Patients may not use the term ‘spirituality’, hence, listening carefully to the patient’s vocabulary is of major importance. See below for alternative ways to ask the patient about their spirituality.

### Multi-disciplinary spiritual care

The EAPC recognises the value of shared learning across disciplines, and this is pertinent to this topic as SC is the responsibility of all staff members according to the Interprofessional Model of Spiritual Care [29], which will be referred to in this paper as a ‘Multi-disciplinary Model of Spiritual Care’. A Multi-disciplinary Model of SC works within the holistic or bio-psycho-social-spiritual model of the human [30] and recognises that all members of a clinical team have responsibility for spiritual care, but may have different levels of expertise. While all team members should be at least a generalist in SC, the healthcare chaplain [31] is the one, who is an expert of SC [32], and where possible, patients with spiritual needs and/or distress should be referred to expert. However, it is recognised that the form which SC takes will be influenced by factors such as the country’s religious history, culture, health care system, local traditions, type of healthcare institution and its organisational culture, and resources available. It is important that communication between staff members is clear so that all are aware of who is providing SC for each patient or caregiver. See Table 1 for discussion of terms to describe aspects of SC provision.

**Table 1:** Terminology

Spiritual care Spiritual care involves recognizing the importance of the spiritual dimension of care for patients and involves needs assessment and provision of support.	
Healthcare SC worker The staff member who has specialised in spiritual care within healthcare institutions, dedicated to SC of patients, regardless of their religious background. May also be called ‘chaplain’, ‘pastoral care worker’, ‘board-certified chaplain’, ‘healthcare SC worker’, ‘SC worker’, ‘priest’ or other term, including humanistic counselors or existential philosophers specialized in SC [31].	
Multi-disciplinary team Palliative care is traditionally practised by a team of healthcare professionals, including doctors, nurses, physiotherapists, speech pathologists, occupational therapists, psychologists, healthcare chaplains, social workers, etc. The combined team is called the multi-disciplinary team, also known as a multi-professional team. Not all disciplines may be represented in all units.	

In some countries, rather than using a SC worker trained for healthcare, SC is the exclusive domain of a member of a religious community, e.g. a priest, whose willingness to provide SC is not guaranteed. Some priests prefer to limit their patient interactions to religious rituals, and some are extremely under-resourced and could not meet the needs of all patients even if they wanted to. Sometimes SC is the domain of volunteers or trained staff members rather than full-time spiritual care staff, which can also lead to gaps and lack of standardisation in provision of SC.

These problems are not insurmountable. In many countries we see a growing awareness among healthcare workers, educators and policymakers, leading to integration of SC in national healthcare and education [33–35]. Not all changes will be possible merely through SC education. Provision of SC needs to be recognized as the responsibility of all staff members. Lobbying administrators, key stakeholders and local politicians and promotion of spiritual leadership may be required to establish SC within an institution [36].

### Spiritual, existential or/and religious

At the outset it is noted that problems continue to arise in many places because spirituality is understood to belong only to religious traditions. In some languages translation of the word ‘spirituality’ is synonymous with ‘religion’. In regions with a strong Roman-Catholic tradition, spirituality is often recognised as the core of religion. This confusion can lead to some patients rejecting SC on grounds of not being ‘religious’. As a result, patient concerns around non-religious meaning-seeking and meaning-making can be neglected. In countries where religious talk is forbidden in the workplace, problems obviously arise if this confusion exists. This raises the question of whether an alternative term, such as ‘existential care’ should be used. It is recommended that each country’s representatives decide the appropriate term to be used in their language to maximise acceptance of SC. However, the EAPC will continue to use the term ‘spirituality’ to remain consistent with the WHO definition of palliative care. This paper supports the idea that “spirituality is universal, deeply personal and individual; it goes beyond formal notions of ritual or religious practice to encompass the unique capacity of each individual. It is at the core and essence of who we are, that spark which permeates the entire fabric of the person and demands that we are all worthy of dignity and respect. It transcends intellectual capability, elevating the status of all of humanity.” [37].

## Methods

Early in 2018, a preliminary draft paper was written by members of the EAPC Spiritual Care reference group inviting comment on the four core elements of SC in palliative care as outlined by Gamondi and colleagues in their recommendations for SC training and education. In summary, these proposed that PC professionals should be able to (a) demonstrate personal reflective practice; (b) integrate the patients’ and families’ spiritual needs in the care plan, (c) provide opportunities for patients and families to express their spirituality, and (d) be conscious of the boundaries that may need to be respected in terms of cultural taboos, values and choices [38]. The preliminary draft paper was circulated to experts from the EAPC SC reference group for feedback. At the second stage (July–August) feedback was incorporated into a second draft paper and experts and representatives of national palliative care organisations were invited to provide feedback and suggest revisions. In January 2019 the final paper was submitted to the board of directors of the EAPC for adoption as an official position paper of the EAPC.

## Results

The preliminary draft was reviewed by ten palliative care and care ethics experts from Europe, South America and Australia. The second draft was reviewed by 13 national experts from Europe, Canada, Australia and South America. The 15 members of the EAPC board approved the white paper in March 2019. The final version incorporated the subsequent criticism, and as a result, the Gamondi competencies [38] were explored and critically revised leading to updated suggestions for SC education in palliative care.

### Demonstrate the reflective capacity to consider the importance of spiritual in one’s own life

The first recommendation for training is development of the reflective capacity of staff to consider the importance of spiritual dimensions in their own lives. Research has shown the importance of personal spirituality of care providers in SC competence and confidence [39, 40]. Spirituality in healthcare is not yet taught universally at undergraduate level, although it is beginning to be introduced into curricula for medicine and nursing. In some places the awareness of spirituality in healthcare and SC depends on a local ‘champion’ who introduces discussions about spirituality, whether there is official recognition of SC or not. The greater the proportion of staff members who are not in touch with their spiritual needs, the more complicated provision of SC becomes, and the less likely that the SC needs of the patients are met. If spiritual needs of patients remain unaddressed, spiritual suffering can ensue [41].

It is noted that high personal spirituality and competence in SC is associated with reduced burnout in palliative care professionals [36, 42]. This is a secondary reason why universal training in SC is recommended for all palliative care staff, and the reason why the Multi-disciplinary Model of Spiritual Care is recommended in the palliative care setting.

While self-reflection on spiritual issues in the individual’s own life should be a standard part of SC training, it was noted that SC is a new concept in many places, and that healthcare professionals have not been in the habit of practising personal spiritual reflection as a professional requirement. Self-awareness can help the healthcare practitioner to avoid being distracted by their own fears, prejudices and restraints and attend to the patient [43].

There are practical challenges in facilitating an intimate process such as self-reflection in a group of healthcare professionals. Solutions include the following. Note that some cost may be involved with some programs:

- The development of regular voluntary informal forums where staff can discuss personal spirituality over a cup of coffee is supportive of self-reflection.
- **Schwartz Rounds** [44] are an evidence-based forum for staff from all backgrounds to come together to talk about the emotional and social challenges of caring for patients. The aim is to offer staff a safe environment in which to share their stories and offer support to one another.
- A **Balint Group** [45] is a group of clinicians who meet regularly to present clinical cases in order to improve and to better understand the clinician-patient relationship, and to provide mutual support.
- **Circle of Trust**, based on the work of Palmer [46, 47], involves group study sessions which focus on creating a space for staff members to listen to themselves and reflect on their SC practices.
- Continuing professional development opportunities to address personal spiritual development, such as spiritual retreats.
- Self-assessment tools:
  The **Spiritual Attitude and Involvement List** (SAIL) [48] is a spiritual well-being scale which has been validated in healthy populations, and defines spirituality broadly on a non-theistic basis. It can also be used for patients.
  The **Spiritual Care Competency Scale** (SCCS) [49] is a validated measure designed to assess nurses’ competencies in providing SC.
  The **Spirituality and Spiritual Care Rating Scale** (SSCRS) [50] is a validated measure designed to establish how nurses perceive spirituality and SC.
  The **Spiritual Care Competence Questionnaire** (SCCQ) [49] quantifies specific SC competencies in different professions. It is a validated scale designed to measure competence in SC for both health professionals and pastoral workers. The SCCQ does not assume that the SC provider is religious. It is available in multiple languages. http://spiritual-competence.net/Questionnaire/
  The **Ars Moriendi** (“Art of Dying”) or Diamond-model [51, 52] is inspired by a medieval tradition to create a framework for reflection and conversation on spirituality in a secular palliative care context for both staff and patients.
- An understanding of the human condition is considered to be a valuable contribution to effective spiritual discussion at the EOL [43, 53], for example through engagement with the humanities e.g. poetry, novels, art etc.

### Recognise the importance of spirituality in the life of the patients, and understand the patients’ and families’ spiritual, existential and religious needs, respecting their choice not to focus on this aspect of care

The second recommendation involves understanding how spirituality impacts the life of the patient. To do this staff need to provide opportunities for patients and families to express the spiritual dimensions of their lives in a supportive manner, and to respect the patient’s beliefs, regardless of one’s own.

Sudden ill-health or significant loss, diagnosis of a chronic or life-limiting illness threatens a patient’s understanding of their world, as they are forced to confront their own limitations and mortality, potentially precipitating an existential crisis [41]. Spirituality becomes important to patients as they approach their own death [21, 54]. Spiritual wellbeing contributes to patients’ quality of life and their ability to cope with terminal illness [55–58], assisting the patient in achieving a sense of well-being at the end of life [59]. Spirituality of the patient should be addressed at all points of the patient’s illness trajectory, and not left until the very end of life. However, while early intervention increases the opportunity for the patient to benefit from SC [60], it is important to note that the patient’s initial ‘no’ may represent vulnerability [61]. Hence the offer of SC should not be a once-only action, but a continuous journey signalling presence, interest in building a relationship, and maintaining a connection.

Training is required to learn how to elicit a spiritual history. Where staff has received approved SC training, the provision of care is superior [40]. Van de Geer and colleagues reported improved healthcare professionals’ attention for spiritual needs and decreased sleeping problems using patient reported outcomes measures [62]. A range of services is usually available to support the patient spiritually in the palliative care setting, although access to resources varies across jurisdictions. Resources which support patient and family spirituality include: SC workers or volunteers; discussion of spirituality during ward rounds or in individual consultations; family meetings [63]; music and art therapy; and symbolic acts in the palliative care unit when a patient has died. These may include turning on a light until the patient’s body has left the ward, or the lighting of candles, or the saying of a blessing when the patient’s room becomes empty. SC should be offered to all patients, however it is recognised that some patients and families want to arrange their own SC. In some units it is possible to bring spiritual carers from the community into the hospital or hospice to support patients and their families.

Barriers to the provision of SC include: lack of knowledge and uncertainty in opening a conversation for this domain. There can be a lack of appreciation of the need for SC, or difficulty in its provision, such as how to provide SC to agnostic or atheist patients [15], the time required for SC; mismatch between the religion of a priest and a patient; sometimes symbolic acts, such as lighting candles, break hospital safety regulations, and substitutes may need to be developed. Note that the empirical evidence suggests that lack of time and belief mismatch do not need to constitute barriers to spiritual care [60].

In order to provide appropriate SC, it is recommended that a spiritual history be taken at the time of admission. Any staff member can do this. The first step of SC constitutes understanding the patient’s spiritual framework and values; the second step involves screening or triage for spiritual problems. Patients with spiritual needs should be referred for expert SC, where the spiritual care worker will administer a detailed assessment. Many models exist for each stage. It is recommended that the purpose of the questioning be considered carefully before a tool is chosen [64, 65].

#### Spiritual history

1. The **Ars Moriendi** (“Art of Dying”) or Diamond-model [51, 52] is inspired by a medieval tradition which creates a common framework for communication and reflection on spirituality in palliative care within a secular and/or multi-faith society.
2. **FICA** [66] is a spiritual history-taking tool which was developed to help health care professionals address spiritual issues with patients in all settings. FICA serves as a guide for conversations in the clinical setting rather than a checklist, and is especially effective for patients who follow an organised religion.
3. The **SPIRITual History** [67] is a guide to identifying important components of the spiritual history for a broad range of patients.
4. **HOPE** [68] is a spiritual history-taking tool developed in a general practice context.
5. **FAITH** [69] is a spiritual history-taking tool which was developed for physicians and medical students.
6. **Q2-SAM** [70] is a model developed to ensure person-centred care in nursing. It is based on two questions: What’s most important to you now? How can we help?

While the wording of these tools may not be comfortable for everyone, it is helpful for beginners to start with a framework for asking questions. Experienced SC providers tend to use their own words to elicit information from the patient about what values are most important to them in the provision of holistic care [61], such as ‘What is it that helps you to cope when things are really difficult?’, or ‘What or who is it that gives meaning to your life?’ [61].

#### Screening for spiritual need

Recommendations include screening all patients for spiritual concerns at the time of admission by a palliative care team member with referral to the SC worker as required. Examples of tools by which to elicit spiritual concerns include:

1. The **JAREL Spiritual well-being Scale** [71] was developed as an assessment tool to establish a nursing assessment of spiritual well-being and is validated.
2. The question ‘**Are you at peace?**’ is a screening tool validated both as a measure of spiritual wellbeing and spiritual suffering [72].
3. There are individual questions about spiritual needs embedded in a number of psychosocial screening tools. Note that many other physical, psychological and social needs are also assessed by these measures. Examples include the **Canadian Problem Checklist** [73], **James Supportive Care Screening** [74], **Distress Inventory for Cancer (version 2)** [75], and the **Advanced Cancer Patients’ Distress Scale** [76].

#### Detailed spiritual assessment

Where a healthcare chaplain is present, a more detailed spiritual history may be used. This may include enquiry about the main spiritual needs of the patient, and which spiritual resources the patient already accesses, so that the healthcare chaplain can work with what already exists. In the absence of a healthcare chaplain, this role may be temporarily filled by another staff member. More detailed history-taking tools include the following:

1. A popular method of assessment is described in ‘**The discipline for pastoral care giving**’ [77].
2. The **PC-7** [78] is an evidence-based, quantifiable model for the assessment of unmet spiritual concerns of palliative care patients near the end of life. It was developed by a team of chaplains working in palliative care.
3. The **7 × 7 Spiritual Assessment Model** [79] aims to assist the process of discerning the spiritual needs of the patient and the resources they already have to help them cope with those problems.
4. The **MD Anderson Spiritual Assessment Model** [80] was developed in a palliative care setting as a way of identifying spiritual distress.
5. **Spiritual AIM** [81] provides a conceptual framework for the chaplain to diagnose an individual’s primary unmet spiritual need, devise and implement a plan for addressing this need through embodiment/relationship, and articulate and evaluate the desired and actual outcome of the intervention.
6. The **Spiritual Distress Assessment Tool (SDAT)** [82] was developed in a setting of care of elderly patients and aims to identify spiritual distress in a clinical setting.

### Integrate the patients’ and families’ and caregivers’ spiritual needs in the care plan and document SC provision

Taking a spiritual history and screening for spiritual need establishes a baseline for each palliative care patient. Some of this information may be recorded in other parts of the patient care plan, such as with social aspects of care or family information. However, all ongoing SC needs to be documented to keep a record of care. Documentation of spirituality and SC in the healthcare setting supports the practice of SC in palliative care during the entire trajectory of illness. Initiatives aimed at standardising vocabulary in SC will facilitate this process [83, 84].

While incorporating SC into the patient’s care plan is encouraged for healthcare staff and SC workers, in many countries it is not currently a routine. There are several reasons for this, including lack of understanding the importance of SC at the end of life, lack of training for staff, lack of vocabulary to describe SC, and absence of standard tools in native languages for spiritual assessment, or a focus on the biological model of the human being only. Also SC workers are not accepted as equals in some palliative care services, and/or need to be specially invited for team meetings. However, there are also situations where SC workers do have access to the medical file and systematically report their activities respecting the patients’ privacy and the ordinary professional restrictions of confidentiality without a problem. SC can be taught so that these problems are overcome [85].

#### Confidentiality

In some places, enquiry about spirituality is seen as very intimate and private concern which should not be broached by healthcare staff. There may be concerns about ethics, or enquiry may be possible only in a more personal environment such as the patient’s home or private healthcare settings. There is also confusion about how confidential spiritual information is. If a SC provider is a member of the healthcare team, they are obliged to communicate any information about the patient which will impact their care. This is because healthcare operates within a multidisciplinary context, and all members of the team rely on each other to convey whatever information they have obtained professionally to work together for the benefit of the patient. Occasionally it may be necessary to ask for the patient’s consent. It is possible that religious care may be given in the context of the confessional, but this does not represent spiritual care in healthcare. If information is particularly sensitive, it may be necessary to get patient consent to communicate it to other staff (either verbally or in the medical record). In places where spiritual care workers are not allowed to write in the patient record, administration should be lobbied to allow this for the benefit of the patient. It is recognised that, for some healthcare chaplains or healthcare workers, a dilemma may exist between the obligation to their role as a member of the healthcare team, and the obligation to their spiritual tradition. The needs of the patient should guide documentation.

### Be conscious of the boundaries that may need to be respected in terms of culture, ritual and traditions

The question of culture and cultural competencies emerges frequently in discussions of palliative care [86–90]) but the evidence on culturally specific needs in palliative care is extremely limited [85, 91], suggesting that this is an area that requires more research.

Patients tend to prefer healthcare structures and systems that make them feel safe and well-served [92]. Respecting patients’ culture-based aspirations may be encouraged and supported as long as they do not challenge the patient’s well-being. In ethical terms, the healthcare provider’s role is to protect patients from harmful culture-bound activities. In order to do so, networking with local communities is essential.

Some patients and families want to arrange care according to their own practice, which may include family visitations, food-related rituals or inviting (religious) leaders from the community into the hospital to support patients and their families. Staff should be trained to welcome this. Pro-active engagement with local (religious) communities, funeral homes etc. to establish the ground rules of ‘what is acceptable’, is recommended to avoid conflict. Networking and open communication in this regard benefits patients and caregivers as well as supporting healthcare staff in care provision.

Culture is a highly context-dependent concept described as ‘a patterned behavioural response that develops over time as a result of imprinting the mind through social and religious structures and intellectual and artistic manifestations’ [88]. Ritual is an example of this, although rituals in palliative care setting can be institutional and not necessarily culture-bound. Ritual is an act that can send a message of belonging and being cared for. If ritual helps patients to cope with illness [94], it is to be supported, however, if the patient agrees to this to please his/her family it should be negotiated carefully, whatever culture is involved.

The culture and traditions of the patient should be kept in mind as the patient care plan is developed. This may include regular recording of cultural information that will impact on advance care planning, dietary requirements, or arrangements after death, as part of the care plan for the patient. All the above should be appropriate for a secular as well as a religious culture, staff being aware of cultural and religious diversity in the local population.

Cultural taboos may not be observed if the staff is not aware of them – this is particularly an issue where new cultural populations, such as refugees, are present. There can be resistance when cultural needs conflict with evidence-based medicine [95]. This can be a source of stress to palliative care staff if it results in increased suffering at the end of life, or alternatively, loss of patient autonomy. Didactic teaching on cultural and religious practices across populations is required in order to improve understanding of comparative religions. All healthcare staff needs to be aware of the risk of prejudice, and training may be needed to promote inclusivity of all cultures within the palliative care service. The Purnell Model for Cultural Competence provides a basis for understanding individual cultural needs in self-reflection and in contact with patients and relatives, in order to provide holistic, cultural competent interventions in healthcare [96]. It can be used in teaching and self-assessment.

#### Staff training

Progression of SC standards in palliative care will only be achieved through widespread and ongoing training of palliative care staff. SC training should be made available in all palliative care units, including opportunities for personal spiritual reflection. Education should be multi-disciplinary and accessible (both academically and financially) to all staff. Training in work time is preferred. Topics to be covered would include the four recommendations central to this paper. National guidelines are already available, for example Germany [97], The Netherlands [98], and Scotland [99].

Regular refresher courses should be available. This may not need to be local. Online resources are available internationally. It is possible that the EAPC could provide a noticeboard for those arranging SC training to advertise online. The EAPC SC reference group plans to develop online training modules for use in on-site training.

## Discussion

This paper outlines a Multi-disciplinary Model of Spiritual Care, adapted from the suggestions made by Gamondi and colleagues [38]. It is recommended for all palliative care services if resources are available. In order to provide SC in the palliative care context, it is recommended that staff develop the skill of self-reflection. Several practices are available to encourage this skill. Staff needs to recognise the importance of spirituality in the life of the patient, and this requires a holistic approach, with the taking of a spiritual history and screening for spiritual need. In the event of spiritual distress, it is recommended that the patient be referred to a SC specialist, that is, a trained healthcare SC worker, for personalised intervention. SC should be integrated into the patient and caregiver care plans, with initial assessment and ongoing interventions recorded clearly in the patient notes. All staff should be on guard to avoid prejudice interfering with universal SC. While it is recognised that not all palliative care services will currently have the resources to reach all these goals, this document is offered as an aspirational level of SC education to be offered to palliative care patients.

### Future directions

The EAPC Spiritual Care reference group will continue to develop templates and tools to support teaching and practice of SC in palliative care, which will be freely available on the EAPC website. Multi-lingual versions will be available. More research needs to be done to determine effective interventions both preventative and in the case of spiritual distress.

## Conclusion

Based on comments and feedback from international experts as well as extensive research literature this paper explores and extends the Gamondi competencies [38] providing updated and critically revised suggestions for spiritual care education in palliative care. The spiritual care competencies for all palliative care providers are accompanied by the best practice models and research evidence at the same time being sensitive towards different development stages of the palliative care services across the European region. This EAPC white paper encourages and facilities high quality, multi-disciplinary, academically and financially accessible spiritual care education to all palliative care staff.

## Data Availability

The data will be aviable upon resonable request. Requests are to be send to Dr. Piret Paal, Paracelsus Medical Private University in Salzburg, Strubergasse 21 5020 Salzburg Austria. E-Mail: piret.paal@pmu.ac.at

## Abbreviations

EAPC: European Association for Palliative Care
PC: Palliative Care
SC: Spiritual Care
UN: United Nations
UNICEF: United Nations Children’s Fund
WHO: World Health Organization
